# Phase Transitions and Physical Properties of the Mixed Valence Iron Phosphate Fe_3_(PO_3_OH)_4_(H_2_O)_4_

**DOI:** 10.3390/ma15228059

**Published:** 2022-11-15

**Authors:** Maria Poienar, Matthias Josef Gutmann, Gheorghe Lucian Pascut, Václav Petříček, Gavin Stenning, Paulina Vlazan, Paula Sfirloaga, Carsten Paulmann, Martin Tolkiehn, Pascal Manuel, Philippe Veber

**Affiliations:** 1 National Institute for Research and Development in Electrochemistry and Condensed Matter, Str. Dr. Aurel Păunescu Podeanu Nr. 144, 300569 Timisoara, Romania; 2 MANSiD Research Center and Faculty of Forestry, Stefan Cel Mare University, 720229 Suceava, Romania; 3 Rutherford Appleton Laboratory, ISIS Facility, Chilton Didcot, Oxfordshire OX11 0QX, UK; 4 Institute of Physics, Academy of Sciences of the Czech Republic, 182 21 Prague 8, Czech Republic; 5 Mineralogisch-Petrographisches Institute, Universität Hamburg, 20146 Hamburg, Germany; 6 Deutsches Elektronensynchrotron DESY, Notkestrasse 85, 22603 Hamburg, Germany; 7 Institut Lumière Matière, Université Claude Bernard Lyon 1, University of Lyon, CNRS, F-69622 Villeurbanne, France

**Keywords:** Fe_3_(PO_3_OH)_4_(H_2_O)_4_ iron phosphate, synthesis, crystal structures, structural and magnetic phase transitions, mixed valence, single crystal x-ray diffraction, neutron powder diffraction, density functional theory

## Abstract

Iron phosphate materials have attracted a lot of attention due to their potential as cathode materials for lithium-ion rechargeable batteries. It has been shown that lithium insertion or extraction depends on the Fe mixed valence and reduction or oxidation of the Fe ions’ valences. In this paper, we report a new synthesis method for the Fe_3_(PO_3_OH)_4_(H_2_O)_4_ mixed valence iron phosphate. In addition, we perform temperature-dependent measurements of structural and physical properties in order to obtain an understanding of electronic–structural interplay in this compound. Scanning electron microscope images show needle-like single crystals of 50 μm to 200 μm length which are stable up to approximately 200 °C, as revealed by thermogravimetric analysis. The crystal structure of Fe_3_(PO_3_OH)_4_(H_2_O)_4_ single crystals has been determined in the temperature range of 90 K to 470 K. A monoclinic isostructural phase transition was found at ~213 K, with unit cell volume doubling in the low temperature phase. While the local environment of the Fe^2+^ ions does not change significantly across the structural phase transition, small antiphase rotations occur for the Fe^3+^ octahedra, implying some kind of electronic order. These results are corroborated by first principle calculations within density functional theory, which also point to ordering of the electronic degrees of freedom across the transition. The structural phase transition is confirmed by specific heat measurements. Moreover, hints of 3D antiferromagnetic ordering appear below ~11 K in the magnetic susceptibility measurements. Room temperature visible light absorption is consistent with the Fe^2+^/Fe^3+^ mixed valence.

## 1. Introduction

Rechargeable batteries are an efficient way to store electrical energy in the form of chemical energy. Thus far, lithium-ion rechargeable batteries are considered to be the most promising candidates [1,2,3,4,5,6]. Recently, iron phosphate materials have been extensively investigated for potential electrode materials in rechargeable batteries due to the discovery of LiFePO_4_ material, which is considered inexpensive and environmentally friendly [7,8,9]. For the case of lithium-ion rechargeable batteries, cathode materials are usually host compounds into/from which lithium ions of the electrolyte can be inserted/extracted as a guest species [10]. It has been shown that insertion of lithium ions into LiFePO_4_ and Li_3_Fe_2_(PO_4_)_3_ occurs through the reduction of Fe^3+^ to Fe^2+^ [11,12]. Therefore, understanding the temperature-dependent behavior of the crystal structures and their influence on the electronic properties is of great interest. Phosphate materials could lead to a new playground for studying the structural, electronic and magnetic properties in order to find materials with improved characteristics for cathode applications. Out of these phosphate materials, the Fe-containing class based on Fe/P/OH (iron hydroxyphosphates) stands out due to the complexity of their crystal structures, which is the result of various compositions with varying water content, leading to a large range of exotic properties [13]. Besides this, iron phosphate materials show additional interesting properties with many possible applications such as catalysis [14,15], electrode materials for Li-ion batteries [15,16,17,18,19], soil fertilization [20] and pigments in art paintings [21].

Iron hydroxyphosphates occur naturally, and their properties have been intensely studied in order to understand how to obtain artificially synthetic phosphate compounds. In this respect, different synthesis routes were used, varying the composition/element ratio/water molecules aiming to obtain novel induced properties [8,14]. Among these routes, hydrothermal synthesis was the most successful in synthesizing the naturally occurring or unknown phases of iron hydroxyphosphates or hydrate phosphates. Although many studies have been dedicated to the structural, magnetic, electronic and catalytic properties of iron phosphate minerals, such as (Fe^2+^)(Fe^3+^)_2_(PO_4_)_2_(OH)_2_ (barbosalite) and (Fe^2+^, Mn^2+^)(Fe^3+^)_2_(PO_4_)_2_(OH)_2_ (lipscombite) [22,23,24,25,26,27,28,29], questions still remain unanswered about the electronic–structural interplay in these compounds. Namely, how the Fe mixed valences control the temperature-dependent properties, how the valence ratio of the Fe ions can be tuned by the synthesis methods [25], etc.

In this paper, we report a new synthesis route for obtaining the synthetic mineral Fe_3_(PO_3_OH)_4_(H_2_O)_4_ [30]. In addition, we report temperature-dependent measurements of the crystal structures, specific heat, magnetic susceptibility and absorption spectra, aiming to probe the occurrence of mixed valence states for the Fe ions and the possible underlying electronic–structural mechanism to complement the scarce studies that are available in the literature [14,15]. First principle calculations, in the form of density functional theory, corroborate the experimental findings and point to some kind of electronic ordering across the structural phase transition. The structural and magnetic properties of (Fe^2+^)_3_(PO_4_)_2_·8H_2_O (vivianite) compound, a related mineral that also occurs naturally and has only Fe^2+^, have been extensively studied, and no structural phase transition occurs upon cooling [31] but a long-range antiferromagnetic order has been found below 8.8 K with neutron diffraction [32]. However, upon exposing the vivianite (Fe^2+^)_3_(PO_4_)_2_·8H_2_O compound to air, the mixed valence metavivianite phosphate hydroxide hydrate (Fe^2+^)_3−x_(Fe^3+^)_x_(PO_4_)_2_(OH)_x_·(8 − x)H_2_O compound is found, which is reported to have a possible charge order of the Fe^2+^/Fe^3+^ ions [33]. In contrast, the mixed valence (Fe^2+^)(Fe^3+^)_2_(PO_3_OH)_4_(H_2_O)_4_ studied in this paper does not occur naturally, and only micron-sized crystals have been obtained thus far by a hydrothermal route reacting vivianite (Fe^2+^)_3_(PO_4_)_2_·8H_2_O with FeCO_3_ in the presence of FePO_4_ and water at a temperature of 220 °C [30].

Thus, the complex chemistry of synthesizing these materials, together with various physical properties of iron phosphates, leads to a rich playground in which to discover novel functional materials for potential applications, such as lithium-ion rechargeable batteries. Our current studies complement the existing studies in understanding the complex interplay of charge, spin and lattice degrees of freedom in this class of materials [14,15].

## 2. Experimental and Theoretical Methods

Fe_3_(PO_3_OH)_4_(H_2_O)_4_ samples were obtained by low-temperature and low-pressure hydrothermal synthesis using vivianite (Fe^2+^)_3_(PO_4_)_2_·8H_2_O and H_3_PO_4_ as starting materials, which is a different synthesis route from the one reported in the literature so far. First, the vivianite (Fe^2+^)_3_(PO_4_)_2_·8H_2_O, material was obtained by the co-precipitation method, starting from (NH_4_)_2_HPO_4_ and FeSO_4_·7H_2_O precursors which were added in distilled water and mixed at 80 °C for 1 h on the magnetic stirrer until the precipitation was complete. The sample was then washed with distilled water and dried in air. Second, the obtained vivianite sample (Fe^2+^)_3_(PO_4_)_2_·8H_2_O, was mixed with 1 mL H_3_PO_4_ and 2 mL distilled water in an autoclave (total volume of 45 mL). The autoclave was sealed and placed in a furnace at 110 °C for 5 days. Third, the resulting yellow-light as-obtained precipitate was filtered, washed with distilled water and air dried at 80 °C, thus obtaining our Fe_3_(PO_3_OH)_4_(H_2_O)_4_ sample. To check the temperature stability of the sample, we performed thermogravimetric measurements using a SETARAM apparatus—LabSys Evo model (Setaram, Caluire, France), in which a typical 20–35 mg amount of sample was heated in synthetic air (debit flow of 27 mL/min) at temperatures up to 750 °C (with a temperature rate of 5 °C/min). To check the sample’s quality and morphology, we recorded scanning electron microscope (SEM) images using a FEI Inspect S microscope (FEI, Hillsboro, OR, USA). To characterize the structural, magnetic and electronic properties of the samples, we performed the following measurements: Single crystal diffraction was carried out at 100 K and 300 K at beamline P24 (PETRA III, DESY, Hamburg, Germany) equipped with a Pilatus3 CdTe 2 M detector and a nitrogen cryostream. In this experiment, a crystal of 100 μm size was mounted on a loop on a spindle and rotated through a range of 360 degrees with an oscillation range of 0.25 degrees and 1 s exposure per frame. A wavelength of 0.5166 Å was used and the detector set to a distance of 110 mm; —Laboratory X-ray single crystal data were recorded in fine temperature steps between 90 K and 470 K on a Rigaku Synergy Xtalab X diffractometer (Rigaku, Wroclaw, Poland) using the Molybdenum source and a nitrogen cryostream. Data were treated using Crysalis Pro [34] and XDS [35]; Powder neutron diffraction data were collected at 300 K on the WISH instrument (Oxfordshire, UK) at the ISIS neutron and muon source [36] on about 0.5 g of powder sample. A Rietveld refinement was carried out using the JANA2020 program. The resulting fits prove the single-phase character of the sample; Specific heat data were recorded using a Quantum Design MPMS (Quantum Design, San Diego, CA, USA), in the 2 K–270 K temperature range; Field-cooled (100 Oe) and zero-field cooled magnetic susceptibility were recorded using the same apparatus with the vibrating sample magnetometry setup; The room temperature diffuse reflectance spectra were obtained in the 250–800 nm wavelength range using a Lambda 950 UV–Vis–NIR Spectrophotometer (PerkinElmer, Waltham, MA, USA) with 150 mm integrating sphere. From the reflectance spectra we obtained the absorption spectra, which is an indirect probe of the electronic structure. First principle calculations based on density functional theory were performed to achieve an insight into the electronic–structural interplay of this material. The calculations were performed with the Wien2k code, which implements a self-consistent all-electrons method to solve the Kohn–Sham equations [37]. The generalized gradient approximation Perdew–Burke–Ernzerhof (GGA-PBE) functional was used for the exchange and correlation potential [38]. Muffin-tin (MT) spheres of radius 1.94, 1.34, 1.00 and 0.54 Bohr were used for the Fe, P, O and H ions, respectively. R_MT_*Kmax was set to 2.57, due to the small spheres chosen for the hydrogen ions. The core and valence states were selected by an energy cutoff of −10 Ry. To obtain the charge density in each self-consistent step, the integration over the Brillouin zone of the high-temperature (HT)/low temperature (LT) unit cell was performed by the tetrahedron method with 72/50 special k-points in the irreducible wedge corresponding to a 12 × 3 × 6/7 × 3 × 7 k-point mesh (250/200 k-points in the full Brillouin zone). To compute the density of states (DOS), integrations over the Brillouin zone were performed for 584/316 special k-points in the irreducible wedge corresponding to a 24 × 6 × 12/13 × 7 × 12 k-points mesh (2000/1200 k-points in the full Brillouin zone). During the self-consistent cycles, the convergence criteria for the HT/LT unit cell were set to 0.00005/0.0005 Ry and 0.001/0.005 e^−^ for the energy and charge, respectively. To compute the projected density of states (PDOS), we used tools developed within the eDMFT code [39,40,41,42,43]. An orthogonal local coordinate system (XYZ) was chosen such that it described the oxygen environment around the Fe ions. Then, quasi-atomic orbitals were constructed within this coordinate system from solutions of the Schrodinger equation (inside the MT sphere) projected to bands in a large hybridization window (−10 eV to 10 eV), with respect to the Fermi energy. A schematic representation of the angular part of these quasi-atomic orbitals (in the local XYZ coordinate system) is given later on for Fe^2+^ and Fe^3+^, respectively. They are labelled by t and e symbols depending on the contributions of the five d real orbitals, xy, xz, yz, x^2^ − y^2^ and z^2^, to their linear combinations. t_1_, t_2_ and t_3_ quasi-atomic orbitals are mostly linear combinations of the xy, xz and yz real orbitals, while the e_1_ and e_2_ quasi-atomic orbitals are mostly linear combinations of the x^2^ − y^2^ and z^2^ orbitals.

## 3. Results and Discussions

**Figure 1:**
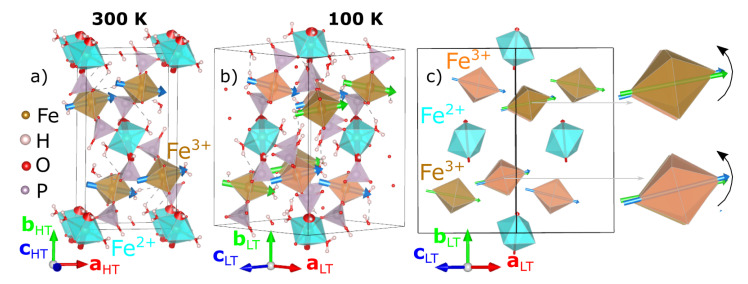
Panels (**a**,**b**), shows the crystal structures at 300 K and 100 K from laboratory X-ray data; Fe^2+^O_6_ and Fe^3+^O_6_ octahedra are colored cyan and beige for the 300 K crystal structure, while they are colored cyan, beige and orange for the 100 K crystal structure; colored arrows are plotted inside the octahedra along the largest Fe-O bonds. Panel (**c**) shows a schematic view of the 100 K crystal structure, showing the three inequivalent octahedra and emphasizing the rotation (black arrow) of the two Fe^3+^ octahedra with respect to the 300 K crystal structure. The relation between the low-temperature (***a****_LT_, **b**_LT_, **c**_LT_*) and room-temperature (***a****_HT_, **b**_HT_, **c**_HT_*) lattice vectors is given by ***a****_LT_ = **a**_HT_ + **c**_HT_, **b**_LT_ = **b**_HT_, **c**_LT_ = −**a**_HT_ + **c**_HT_*.

The room temperature crystal structure of Fe_3_(PO_3_OH)_4_(H_2_O)_4_ determined in this study is shown in Figure 1a. For the HT crystal structure, we are using the same crystallographic settings for the space group and the unit cell as the one used previously in the literature [30].

**Figure 2:**
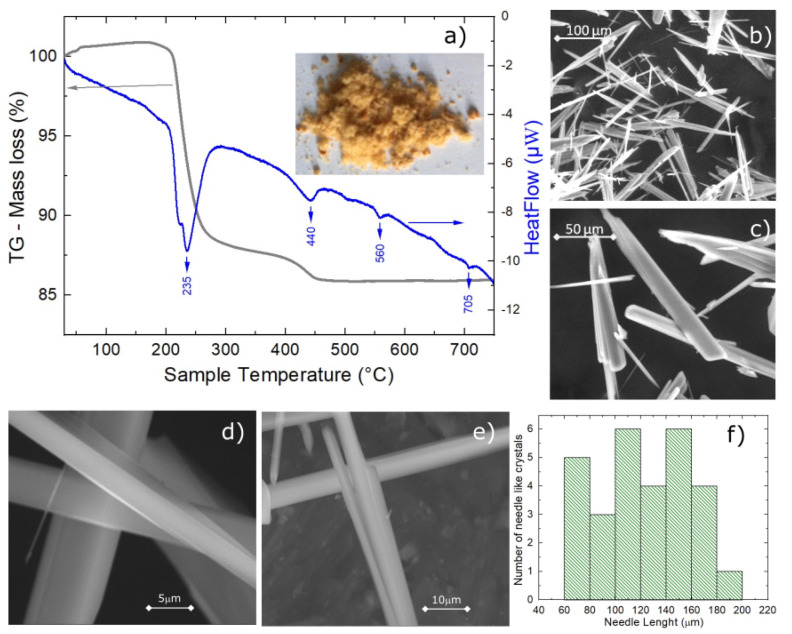
Panel (**a**) TG measurements, indicating the weight evolution, between 30 °C and 750 °C (303 K to 1023 K). The grey line and arrow refer to the TG and the blue line and arrows to the HeatFlow, respectively. The inset photo shows the sample. Panels (**b**–**e**) SEM image for Fe^2+^Fe^3+^_2_(PO_3_OH)_4_(H_2_O)_4_ sample at different magnifications. Panel (**f**) is the single crystal length distribution chart.

Sample stability was checked by the thermogravimetric analysis (TGA) and differential thermal analysis (DTA) in the 30–750 °C (303–1023 K) temperature range. The TGA and DTA curves, shown in Figure 2a, reveal that this compound is thermally stable in a low temperature range, up to approximately 150–200 °C, which is consistent with previous reports for this material [30] and reports for other phosphates, such as the related vivianite mineral [44]. Two weight loss events are observed from the TGA and DTA curves. The first weight loss starts from approximatively 200 °C, as shown by the sharp drop in the TGA and an intense dip in the DTA endothermic curves. The second weight loss looks like a continuation of the first, extending from 270 °C to about 450 °C, as shown by the smooth drop in the TGA curve and the broad dip in the DTA curve. The first/second measured weight loss event corresponds to about 3.7 mg (11.2%)/0.93 mg (2.2%) of water molecules from a starting mass of 33.4 mg. Based on the experimental weight loss, we propose that four and one water molecules, respectively, are sublimated with increasing temperature, according to the following pyrolytic reactions:

235 °C   Fe^2+^Fe^3+^_2_(PO_3_OH)_4_(H_2_O)_4_    Fe^2+^Fe^3+^_2_(PO_3_OH)_4_ + 4H_2_O (1)

440 °C   Fe^2+^Fe^3+^_2_(PO_3_OH)_4_                Fe^2+^Fe^3+^_2_(P_4_O_15_H_2_) + 1H_2_O (2)

**Figure 3:**
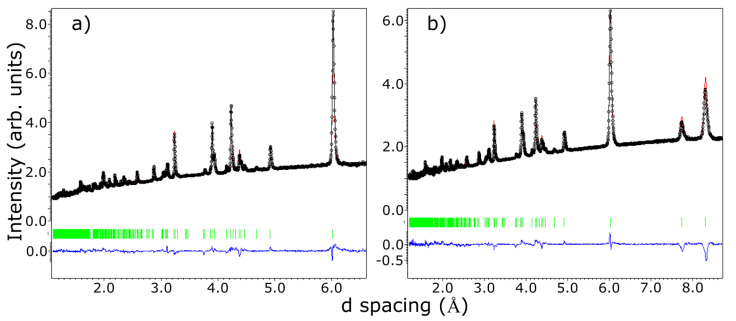
Neutron diffraction measurements collected on WISH instrument at the ISIS spallation neutron source; black open circles represent data collected on bank 3 (2θ = 90 degrees) in panel (**a**) and bank 2 (2θ = 58 degrees) in panel (**b**); red solid line represents the results of the Rietveld refinement; blue solid line represents the difference between the model and the experimental data; green vertical lines represent the position of the d spacings corresponding to crystallographic planes in the crystal model.

**Figure 4:**
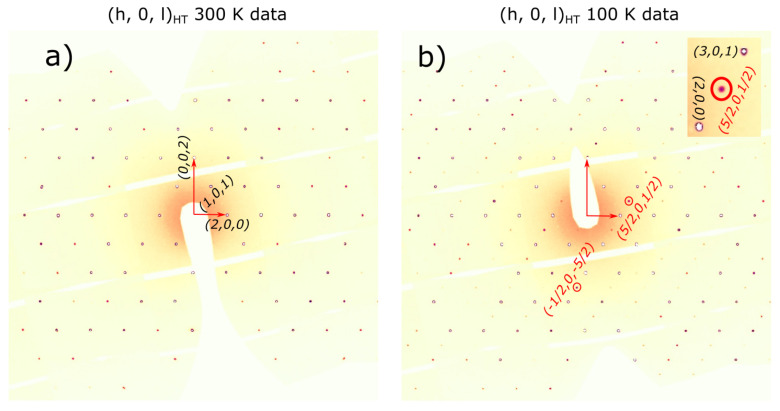
Reconstructed (h 0 l) reciprocal space section at 300 K in panel (**a**) and 100 K in panel (**b**) from the data collected at beamline P24. Several superlattice reflections are marked by red circles; the inset shows the zoom in for one of the superlattice reflections.

**Figure 5:**
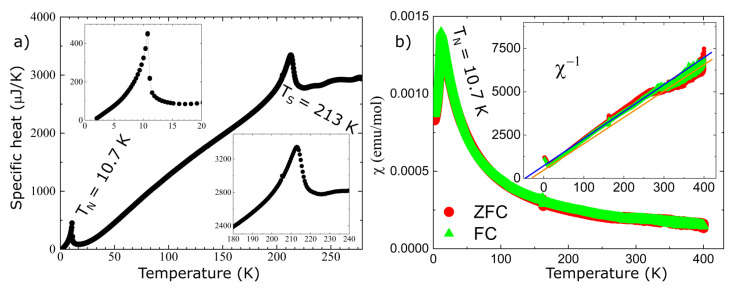
Panel (**a**) shows the specific heat as a function of temperature; the two insets highlight the low temperature region around T_N_ and high temperature region around T_S_, respectively, where T_N_ and T_S_ are the temperatures where we have the magnetic and structural phase transitions; panel (**b**), shows the magnetic susceptibility versus temperature in zero field cooled (ZFC, red symbols) and field cooled (FC, green symbols) mode (H = 100 Oe); the inset shows the inverse magnetic susceptibility data together with two solid lines representing linear fits of the Curie–Weiss behavior, to low and high temperature regions.

The agreement between the measured and calculated weight losses (3.7 mg and 0.93 mg) corresponding to the sublimation of exactly four and one water molecules implies a stoichiometric sample. Moreover, the two weight losses of water molecules are also consistent with the crystallographic structure at HT, which has two inequivalent Fe sites corresponding to the Fe^2+^ and Fe^3+^ ions, each with its own attached water molecules, and thus different activation energies. Besides the two main water losses, we also observe, in the DTA curve, the presence of two small dips around 560 °C and 705 °C with no changes in the TGA curve (thus, no weight loss), suggesting that at these temperatures, there are atomic rearrangements in the sample, consistent with previous reports [30]. SEM images, shown in Figure 2b to e, reveal crystals characterized by a needle-like shape with lengths of 50–200 μm and different widths. The particle size distribution chart that we obtained, shown in Figure 2f, does not have trends of a particular distribution, but if we assume a Gaussian distribution, we obtain a mean particle size of 125 μm with a standard deviation of 36 μm. The reason for the needle-like shape of the single crystal could be the pH of the solution used in the synthesis. The effects of the pH solution on the size and shape of the single crystal were demonstrated for calcium phosphate [45,46,47] and hydroxyapatite [48,49] crystals. Further studies are needed to understand the effects of the pH on the size and morphology of the iron phosphate’s crystals. Neutron powder diffraction does not show any impurity peaks, thus demonstrating the high quality of our sample. A Rietveld refinement of the data was successfully performed using the room temperature crystal structure model (see Figure 3). Using single crystal x-ray diffraction data recorded on beamline P24 at 100 K and 300 K, we found that the HT crystal structure was best described by the monoclinic space group P2_1_/n with lattice parameters a = 8.7512(12) Å, b = 16.6203(29) Å, c = 5.1560(10) Å and β = 90.865(14)° [30]. However, at 100 K, additional reflections were seen at positions corresponding to (½, 0, ½) with respect to the HT cell, as shown in Figure 4. The LT data could be indexed in an enlarged monoclinic cell with a = 10.1057(25) Å, b = 16.5549(23) Å, c = 10.2414(14) Å and β = 119.423(7)°, corresponding to a doubling of the cell volume with respect to the HT cell. A schematic representation of the HT and LT crystal structures together with the crystallographic axes is shown in Figure 1. The LT crystal structure was solved in space group P2_1_/c using the JANA2020 program [50] and Superflip [51]. The validity of the space group was confirmed from the group–subgroup analysis using the Bilbao Crystallographic Server [52]. Hydrogens were located from the Fourier difference maps and included in the refinements using a riding model with the usual constraints on distance and displacement parameter. Refinement statistics are listed in Table 1. Given the two different cells suggesting a temperature-dependent phase transition, a more extended dataset was thus collected on a laboratory X-ray diffractometer to locate the structural phase transition. The occurrence of these superlattice reflections, shown in Figure 4, was monitored as a function of temperature, and they were found to appear below 211(2) K. Additionally, the specific heat curve, shown in Figure 5a, shows a cusp at T_S_ = 213(2) K, confirming the structural phase transition. The structural parameters of the two crystal structures at 300 K and 100 K are given in Table 2 and Table 3.

The crystal structures at 300 K and 100 K are illustrated in Figure 1 using VESTA [53]. Previously reported Mössbauer spectroscopy measurements at room temperature found that the two nonequivalent Fe sites have different oxidation states. Thus, the FeO_6_ octahedra have been colored to highlight the distribution of Fe^2+^ and Fe^3+^ sites (Fe^2+^O_6_—cyan and Fe^3+^O_6_—beige and orange, depending on the HT or LT crystal structure). At 300 K, the crystal structure, shown in Figure 1a, consists of layers of octahedra perpendicular to the b-axis, with the Fe^2+^O_6_ layers alternating with wrinkled Fe^3+^O_6_ layers. Within the Fe^2+^O_6_ layers, the octahedral tilts (red arrows) are aligned in the bc-plane, oscillating around the *b*-axis. Within the wrinkled Fe^3+^O_6_ layers, the octahedral tilts (blue arrows) are aligned in the ac-plane, oscillating around the *a*-axis.

The FeO_6_ octahedra are neither corner- nor edge-shared but rather linked together by PO_4_ tetrahedra. The bond lengths and bond-valence sums within the FeO_6_ octahedra, shown in Table 4, were obtained using the program VESTA [54] and bond-valence parameters from [55] using R_0_ = 1.734 Å and b = 0.37 Å. The Fe^2+^O_6_ octahedra have pairs of long (Fe_1_^2+^–O(3) ~2.213 Å), medium (Fe_1_^2+^–O(5) ~2.155 Å) and short (Fe_1_^2+^–O(10) ~2.065 Å) Fe–O bonds forming the three principal axes of the octahedron. At the corners of the octahedron, for the longest Fe-O bond, there is an associated OH group (O(3)–H1_O3_ bond), for the medium Fe-O bond, there is an associated H_2_O water molecule (O(5)–H1_O5_ and O(5)–H2_O5_ bonds), while, for the shortest Fe-O bond, there are no H ions or H_2_O molecules attached to the oxygen. In previous reports, this environment was described as the hydrogen having a donor–acceptor character, donating or removing charge from the octahedron [30]. The apical hydrogen, H1_O3_, is shared with a neighboring PO_4_ tetrahedron. The bonds within the Fe^3+^O_6_ octahedra are generally shorter than in the Fe^2+^O_6_ octahedra, and they are all distinct in the range of ~1.924 Å to ~2.084 Å, giving the octahedron a slightly more irregular shape. In both octahedra, the longest bonds (Fe_1_^2+^–O(3) ~2.213 Å x2) for Fe^2+^O_6_ and (Fe_2_^3+^–O(9) ~2.084 Å x1) for Fe^3+^O_6_ are associated with water molecules at octahedra corners. For the Fe^3+^O_6_ octahedra, along the direction of the longest bond Fe–O (Fe_2_^3+^–O(9) ~2.084 Å), at the opposite corner of the water molecule (Fe_2_^3+^–O(4) ~1.994 Å), there is no apical hydrogen shared between O(4) and the PO_4_ tetrahedron. This is different from the case of the Fe^2+^O_6_ octahedra where water molecules are found at both ends of the longest Fe-O bonds. Bond-valence sums, given in Table 4, suggest mixed valence for the Fe ions in the Fe_3_(PO_3_OH)_4_(H_2_O)_4_ compound, with the Fe^2+^ and Fe^3+^ oxidation states consistent with earlier reported Mössbauer spectra [30]. 

**Figure 6:**
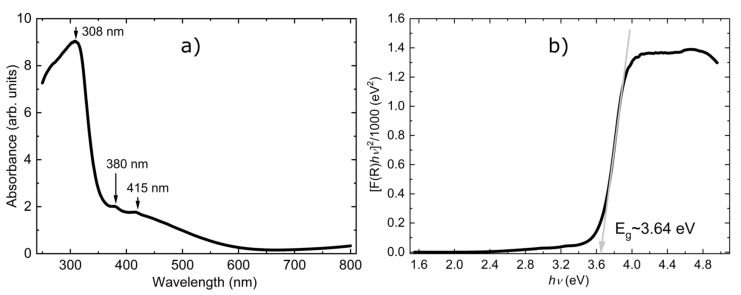
Panel (**a**) UV–Vis absorption spectra in the 250–800 nm range; the arrows show the three optical bands at 308, 380 and 415 nm. Panel (**b**) Tauc plot for the direct bandgap; the gray arrow shows a fitted line to the linear part of the curve; the intercept of the gray solid line with the energy axis defines the value of the band gap.

Further, we focused on the electronic properties and discuss the absorption (F) spectra, which were obtained from the corresponding reflectance (R) spectra by applying the Kubelka−Munk function [56]. The optical absorption spectrum in the UV–visible region (250–800 nm), shown in Figure 6, has three absorption peaks: an intense and broad peak around 308 nm followed by two smaller peaks around 380 nm and 415 nm which indicates a visible-light absorption ability for the Fe_3_(HPO_4_)_4_(H_2_O)_4_ mixed iron phosphate compound. The mixed valence character is also inferred from the similarities of the absorption spectra (similar trends in position and strength of the absorption peaks) with the spectra of the mixed valence barbosalite (Fe^2+^)(Fe^3+^)_2_(PO_3_OH)_2_(OH)_2_. We would like to mention that various mechanisms are reported in the literature to explain similar absorption spectra for mixed valence compounds. Some of them are based on the idea of polaronic transfer (intervalence charge transfer, as the electron transfer between Fe^2+^ and Fe^3+^ may be mediated by O^2−^ ions) [57,58,59], while other mechanisms have their origins in the Fe^3+^ d-d transitions [60]. Thus, bond-valence, added together with the absorption spectra and previously reported Mössbauer spectra at HT [30], is consistent with the mixed valence character of type (Fe^2+^)(Fe^3+^)_2_(PO_3_OH)_4_(H_2_O)_4_. The applicability of the mixed valence iron barbosalite Fe_3_(PO_4_)_2_(OH)_2_ compound for visible light absorption is shown in [26,61,62]. Owing to the similarities in properties with the barbosalite Fe_3_(PO_4_)_2_(OH)_2_ compound, we expect that Fe_3_(PO_3_OH)_4_(H_2_O)_4_ could also have potential applications for visible light absorption.

For a better presentation of the macroscopic measurements of this material, we also extracted the optical gap using the Tauc method [61,62]. This method assumes that the energy-dependent absorption coefficient F(R) can be expressed in terms of the incident photon energy (hυ) and the band gap energy E_g_ by the following expression [F(R) hυ]^n^ = C (hυ-E_g_), where C is a constant and n is a coefficient whose value is determined by the type of electronic transition giving rise to the absorption and can take the values 2 or ½ for direct and indirect band gaps, respectively. Figure 6b shows the reflectance data transformed according to the expression above. The spectra show a linear regime (steep linear change) which is characteristic for wide band gap semiconductors. The intersection point between the extrapolation of the linear fit of the Tauc plot with the energy axis offers an estimate of the optical band gap energy which, in this case, is E_g_ ~ 3.64 eV (here, we assume that the gap is a result of a direct electronic transition).

**Figure 7:**
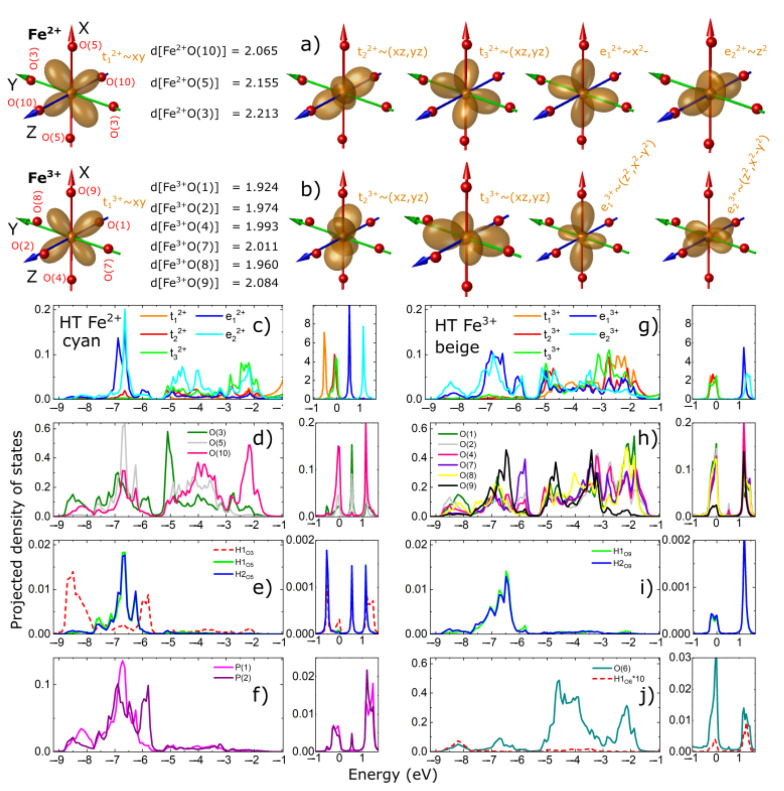
Projected density of states for the atoms of the HT unit cell: Panels (**a**,**b**) show a schematic representation of the XYZ Cartesian coordinate system constructed to best fit the directions of the oxygen ions; the oxygen ions are represented by the red spheres and labeled according to Table 2 and Table 3; the t and e orbital are labeled by 2^+^ or 3^+^ depending on whether they correspond to Fe^2+^ or Fe^3+^. For each orbital, we show the contributions from the real orbitals, for example, t_2_^2+^~(xz, yz) means the second orbital of the Fe^2+^ for which the t_2_ orbital is a linear combination of the real xz and yz orbitals. Panels (**c**–**j**) show the projected density of states for the Fe-d, O-p, P-p and H-s atoms. Fermi energy is represented by the 0 value on the energy axis.

In order to obtain qualitative insights into the microscopic properties, we have performed nonmagnetic calculations for the HT unit cell. The results are presented in Figure 7 as orbital projected density of states (PDOS). In each panel, the PDOS is plotted in two energy panels, one around the Fermi energy (−1.04 eV to 1.7 eV) and the other below the Fermi energy (−9.2 eV to −0.96 eV), to point out the different scales of DOS. As discussed in Section 2, the Fe^2+^ and Fe^3+^ d-PDOS are labeled by t_1_, t_2_, t_3_, e_1_ and e_2_. A schematic representation of these quasi-atomic orbitals given in the local orthogonal coordinate XYZ system of the oxygen ions can be seen in Figure 7a,b for Fe^2+^ and Fe^3+^, respectively. Looking at the PDOS plots, we can identify three energy regions depending on the strength of DOS. The first region around the Fermi energy, between 1.7 and −1.0 eV, where the largest contribution comes from the Fe d−PDOS, the second region, between −1 eV and −5.5 eV, where the largest contribution comes from the O p−PDOS, and the third region, between −5.5 eV and −9.2 eV, where the largest contribution comes from the P/H p/s−PDOS. Of course, due to hybridization between the Fe−d, O−p, P−p and H−s states, we find some contributions of all PDOS states in the three energy regions. From Figure 7c,d for Fe^2+^ and Figure 7g,h for the Fe^3+^ ions, we see that the t−type orbitals have equally strong hybridization with the oxygen ions in the second energy region while the e–type orbitals hybridize stronger in the third energy region. This is expected if we look at Figure 7a,b, where we see that the e–type orbitals are pointing directly towards the corners of the octahedra and thus, towards the oxygen ions, while the t–type orbitals are pointing towards the face of the octahedra, thus, in between the oxygen ions. We also observed that the crystal field significantly splits the t−type orbitals for Fe^2+^, while for Fe^3+^, they look almost degenerate (see Figure 7c,g) in the first energy region. In terms of orbital occupation, we see that for Fe^2+^, the t_1_ orbital is fully occupied, the t_2_ and t_3_ orbitals are mostly occupied and the e_1_ and e_2_ orbitals are slightly occupied due to the covalent bonding with the O, P and H ions due to hybridization. For Fe^3+^, all t orbitals are mostly occupied and the e orbitals slightly occupied due to the covalent bonding. Thus, we see that t orbitals have a higher occupation for the Fe^2+^ than for Fe^3+^, which is qualitatively consistent with the mixed valence scenario.

Now, we turn our attention to the LT crystal structure, shown in Figure 1b. The structural phase transition below T_S_ consists of the sudden rotation of the Fe^3+^ octahedra accompanied by slight distortions of the Fe-O bonds. The Fe^2+^ site remains a single crystallographic site within the LT unit cell (Fe_2_ in Table 3), whilst the Fe^3+^ site splits into two inequivalent sites (Fe_1_ and Fe_3_ in Table 3). These are colored as cyan (Fe^2+^O_6_) and beige/orange for the (Fe^3+^O_6_). We first discuss the individual octahedra. The bond-lengths and bond-valence sums within the FeO_6_ octahedra at 100 K are listed in Table 4. The Fe^2+^O_6_ octahedra distorts such that all six Fe-O bonds are of different lengths. These can still be grouped into long (Fe_2_^2+^–O(3) ~2.192 Å and Fe_2_^2+^–O(1) ~2.185 Å), medium (Fe_2_^2+^–O(12) ~2.162 Å and Fe_2_^2+^–O(16) ~2.139 Å) and short (Fe_2_^2+^–O(20) ~2.075 Å and Fe_2_^2+^–O(18) ~2.062 Å) bonds with their associated presence or absence of hydrogen. In the two LT inequivalent Fe^3+^O_6_ octahedra, the Fe-O bond associated with a water molecule shortens/elongates in the beige (Fe_1_^3+^–O(17) ~2.060 Å)/orange (Fe_3_^3+^–O(15) ~2.120 Å) octahedra, respectively, when compared with the corresponding Fe-O bond of the HT phase (~2.084 Å). It appears that the orange Fe^3+^O_6_ octahedra are becoming a bit more regular with respect to their bond lengths, whilst the distortions remain similar in the beige octahedra compared to the 300 K Fe^3+^O_6_ octahedra. Next, we discuss the octahedral arrangements within the lattice. The Fe^2+^O_6_ slabs remain planar as in the HT phase, whilst, within the wrinkled Fe^3+^O_6_ layers, the octahedra splits into two inequivalent octahedra (orange and beige) due to rotations, as shown by the black arrows in Figure 1c.

**Figure 8:**
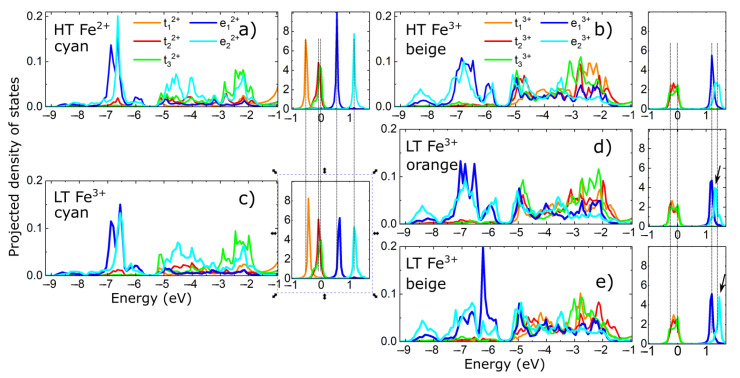
Projected density of states for the Fe d- orbitals: Panels (**a**,**b**) show the Fe–d projected density of states for the HT unit cell; panels (**c**–**e**) show the Fe–d projected density of states for the LT unit cell. Fermi energy is represented by the 0 value on the energy axis; panels (**a**,**c**) have the same legend; panels (**d**,**e**) also have the same legend as panel (**a**). Dotted vertical lines show the correspondence between the peaks in the PDOS computed for the HT and LT unit cell; black arrows in panels (**d**,**e**) show the opposite energy shifts in the LT e_2_^3+^ PDOS peak’s position with respect to the HT PDOS peak.

To understand the effects of distortions in the Fe^3+^O_6_ octahedra in the LT phase on the electronic properties, we have performed additional calculations using the crystal structure outlined in Table 3. By carefully analyzing the PDOS for all the atoms in the LT unit cell, we could again identify three energy regions (depending on the strength of DOS) with similar trends in the hybridization of the states in the three energy regions, similar to the calculations for the HT. Fe d-PDOS are presented in Figure 8c–e. For comparison purposes, in Figure 8a,b, we again plot Fe d–PDOS calculated for the HT. Comparing the PDOS for the Fe^2+^ ions in the two temperature regimes, we observe that there are very small changes in the energy position and shape of the main PDOS peaks, which is consistent with the small structural distortions of the Fe–O bonds within the local Fe^2+^O_6_ octahedra (see Table 4). However, we find visible changes in the energy position and shape for the Fe^3+^ DOS compared with the HT DOS. With increasing/decreasing the Fe-O bond along the direction of the e_2_^3+^-type orbital, its PDOS energy position is decreasing/increasing (see Figure 8d,e) compared with panel b, as expected, for example, in the case of a Jahn–Teller distortion. The electronic configuration of the Fe^3+^ ions is d^5^, and the local environment at HT is already a distorted octahedron, thus, Jahn–Teller-like distortions are probably not the reason for the LT structural distortions. The similarity between the HT- and LT-PDOS for the Fe^2+^ and Fe^3+^ ions give us another indication that, at low temperature, we still have mixed valence of Fe ions (in addition to the bond valence sums). The pattern of the structural distortions at LT, together with the mixed valence character of Fe ions, points to some unusual electronic states in the Fe_3_(PO_3_OH)_4_(H_2_O)_4_ compound. We see similarities with other systems such as AgNiO_2_, where a pattern of structural distortions exists at LT and is associated with a charge-disproportionation inside a metallic state [63,64], BiMnO_3_ and LaMnO_3_, where a pattern of structural distortions exists at LT and is associated with an orbital order that can be interpreted as site and orbital selectivity [65,66,67,68,69], RNiO_3_, where a pattern of structural distortions exists at LT and is associated with a metal to insulator transition [70,71,72,73], TM_2_Mo_3_O_8_ (TM = Mn, Fe, Co, Ni, Zn), where tiny structural distortions exist together with band and Mott gaps within the same electronic state [74,75], etc. Thus, our work shows that further theoretical and experimental studies are necessary to obtain deeper insights into the electronic–structural interplay responsible for the structural phase transition discovered in the Fe_3_(PO_3_OH)_4_(H_2_O)_4_ compound.

Other macroscopic properties such as specific heat and magnetic susceptibility have been measured. At lower temperature, both specific heat and magnetic susceptibility show the onset of long-range magnetic order at around T_N_~11 K, as shown in Figure 5. However, no peak is seen in the magnetic susceptibility at the structural transition, implying that this is a purely structural transition. Combining all the information, we suggest that the structural phase transition is most likely induced by the electronic instabilities of the Fe^3+^ ions. The inverse susceptibility shows two linear regimes in the paramagnetic state, spanning between 40 K–250 K and 250 K–300 K. The Curie–Weiss fits in these regimes result in Curie temperatures of approximately −50 K and slightly lower, both consistent with antiferromagnetic ordering. The Curie–Weiss behavior is consistent with the presence of a local moment on the Fe sites, which shows the existence of strong correlations between the d electrons of the Fe ions. Thus, to obtain quantitative results, a more appropriate theoretical method should be used to investigate this material, such as density functional theory plus embedded dynamical mean field theory [39,40,41,42,43]. Although the theoretical method used in this paper is good to obtain qualitative results about the electronic properties, it cannot describe the presence of local moments on the Fe ions in the paramagnetic state or the existence of a gap, as demonstrated by the Tauc plot.

**Figure 9:**
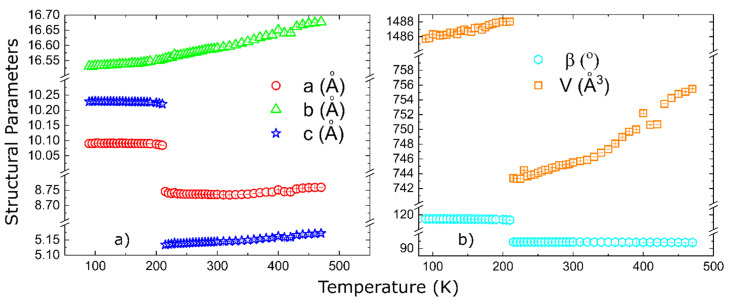
Temperature dependent lattice parameters: panel (**a**) shows the lattice parameters, while panel (**b**) shows the monoclinic angle and the unit cell volume.

For a complete characterization of the structural transition, we also present the temperature-dependent evolution of the structural parameters. They evolve smoothly, above and below the phase transition, as shown in Figure 9. Powder neutron diffraction experiments are underway to solve the magnetic structure. The Supporting Information contains an animation of the crystal structure as a function of temperature, with the crystal structure projected along the three principal crystallographic axes with respect to the LT cell.

## 4. Conclusions

In this paper, we propose a new route to synthesize the mixed valence mineral (Fe^2+^)(Fe^3+^)_2_(PO_3_OH)_4_(H_2_O)_4_. We have performed a series of microscopic and macroscopic measurements such as single crystal x-ray diffraction, specific heat, magnetic susceptibility and optical reflectance. Temperature dependent x-ray measurements showed the presence of an isostructural monoclinic phase transition at T_S_~213 K, with the HT crystal structure being described by the monoclinic P2_1_/n setting, while the LT crystal structure (with a doubled volume) was described by the P2_1_/c setting. Specific heat and magnetic susceptibility measurements confirm the presence of the structural transition at T_S_ and, in addition, they reveal the presence of a magnetic transition at lower temperatures (T_N_~11 K). HT reflectance spectra, plotted as the Tauc plot, give an optical energy gap of 3.64 eV. Theoretical calculations, within the density functional theory, performed for both the HT and LT crystal structures, are presented in the form of orbital projected density of states. We find a correspondence between the orbital projected density of states and the distortions of the Fe octahedra, which implies some kind of electronic order, for example, orbital or charge order, on the Fe^3+^ sites. We also discuss the qualitative hybridization of the Fe-d, P-p, O-p and H-s states.

In conclusion, we have studied the structural properties of the mixed valence mineral (Fe^2+^)(Fe^3+^)_2_(PO_3_OH)_4_(H_2_O)_4_ using single crystal x-ray diffraction, powder diffraction, specific heat and magnetic susceptibility measurements, and we reveal, for the first time, the presence of a phase transition which, we believe, is a consequence of the Fe^2+^/Fe^3+^ mixed valence and a complex electronic–structural interplay. Experimental results corroborated by first principle calculations suggest that the mechanism responsible for the structural phase transition is some kind of electronic order. Similar transitions may exist in other mixed valence iron phosphates and could have an impact on the electrochemical performance of iron phosphates used as cathode materials.

## Figures and Tables

**Table 1:** Data collection statistics at 300 K and 100 K from the P24 and Rigaku Oxford Diffraction instruments; 1st column provides the name of the physical quantities used to described the experiment and data analysis, 2nd and 3rd columns provide the values to the physical quantities from the P24 instrument, while the 4th and 5th columns list the values from the laboratory instrument.

Instrument	P24	P24	Rigaku Oxford Diffraction Xtalab Synergy S	Rigaku Oxford Diffraction Xtalab Synergy S
Empirical formula	Fe_3_P_4_H_12_O_20_	Fe_3_P_4_H_12_O_20_	Fe_3_P_4_H_12_O_20_	Fe_3_P_4_H_12_O_20_
Formula weight	623.5	623.5	623.5	623.5
Temperature	300(4) K	100(2) K	300(4) K	100(2) K
Wavelength	0.5166 Å	0.5166 Å	0.71073 Å	0.71073 Å
Crystal system	Monoclinic	Monoclinic	Monoclinic	Monoclinic
Space group	P2_1_/n	P2_1_/c	P2_1_/n	P2_1_/c
Unit cell dimensions	a = 8.7512(12) Å b = 16.6203(29) Å c = 5.1560(10) Å β = 90.865(14)°	a = 10.1057(25) Å b = 16.5549(23) Å c = 10.2414(14) Å β = 119.423(7)°	a = 8.7363(1) Å b = 16.5905(2) Å c = 5.1443(1) Å β = 90.8860(13)°	a = 10.0916(2) Å b = 16.5328(2) Å c = 10.2303(2) Å β = 119.449(3)°
Volume	749.8(2) Å^3^	1492.3(5) Å^3^	745.524(19) Å^3^	1486.31(6) Å^3^
Z	2	4	2	4
Density (calculated)	2.7616 g/cm^3^	2.7752 g/cm^3^	2.7776 g/cm^3^	2.7864 g/cm^3^
Absorption coefficient	1.337 mm^−1^	1.343 mm^−1^	3.425 mm^−1^	3.436 mm^−1^
F(000)	620	1240	620	1240
Crystal size	-	-	0.32 × 0.06 × 0.05 mm^3^	0.32 × 0.06 × 0.05 mm^3^
Theta range for data collection	1.78 to 43.09°	1.68 to 43.1°	2.46 to 37.565°	2.33 to 37.88°
Index ranges	−22 ≤ h ≤ 22 −43 ≤ k ≤ 43 −7 ≤ l ≤ 8	−25 ≤ h ≤ 26 −43 ≤ k ≤ 43 −23 ≤ l ≤ 24	−15 ≤ h ≤ 14 −28 ≤ k ≤ 28 −8 ≤ l ≤ 8	−17 ≤ h ≤ 17 −28 ≤ k ≤ 28 −17 ≤ l ≤ 17
Reflections collected	30,685	64,524	32,669	64,953
Independent reflections (I > 3σ(I)/all)	7710/8114	14,661/17,688	3261/3863	5263/7690
R(int)	0.071	0.0645	0.0519	0.0571
Absorption correction	Empirical	Empirical	Numerical Gauss Integration	Numerical Gauss Integration
Max. and min. transmission	–	–	1.0 and 0.41	1.0 and 0.47
Refinement method	Full-matrix least-squares on F2	Full-matrix least-squares on F2	Full-matrix least-squares on F2	Full-matrix least-squares on F2
Data/constraints/parameters	8114/24/125	17,688/48/245	3863/24/124	7690/48/244
Goodness-of-fit on F2 (I > 3σ(I)/all)	2.49/2.58	2.48/2.30	2.03/1.90	1.83/1.62
Final R-indices (I > 3σ(I))	R1 = 0.0438 wR2 = 0.1470	R1 = 0.0437 wR2 = 0.1449	R1 = 0.0301 wR2 = 0.0865	R1 = 0.0310 wR2 = 0.0881
Final R-indices (all data)	R1 = 0.0459 wR2 = 0.1564	R1 = 0.0509 wR2 = 0.1475	R1 = 0.0368 wR2 = 0.0885	R1 = 0.0497 wR2 = 0.0951
Largest diff. peak and hole	1.47 and −0.90 e/Å^3^	1.24 and −1.25 e/Å^3^	0.66 and −1.07 e/Å^3^	0.85 and −1.19 e/Å^3^

**Table 2:** Fractional coordinates at 300 K from laboratory X-rays. H atoms were constrained using a riding model with u_iso_ extended by a factor 1.2 of its neighboring non-H atom.

Atom	x	y	z	u_iso_ (Å^2^)
Fe^2+^(1)	½	½	½	0.010950(9)
Fe^3+^(2)	0.13144(3)	0.70271(2)	0.24642(5)	0.00720(6)
P(1)	0.19493(5)	0.57930(3)	0.73474(9)	0.00826(11)
P(2)	0.50621(5)	0.69413(3)	0.25517(9)	0.00642(10)
O(1)	0.14448(18)	0.63823(8)	0.9367(3)	0.0160(4)
O(2)	0.61151(16)	0.72297(8)	0.0436(3)	0.0114(3)
O(3)	0.48438(16)	0.59961(8)	0.2148(3)	0.0108(3)
O(4)	0.35094(15)	0.73528(9)	0.2366(3)	0.0145(4)
O(5)	0.30667(16)	0.44317(7)	0.3103(3)	0.0223(5)
O(6)	0.1022(2)	0.49977(9)	0.7934(4)	0.0303(6)
O(7)	0.57932(17)	0.70142(7)	0.5228((3)	0.0114(3)
O(8)	0.15868(18)	0.60588(8)	0.4606(3)	0.0146(4)
O(9)	−0.10176(15)	0.67714(8)	0.2636(3)	0.0180(4)
O(10)	0.36376(17)	0.55770(9)	0.7667(3)	0.0162(4)
H1_O3_	0.46424	0.58572	0.06122	0.0130
H1_O5_	0.27385	0.46079	0.17132	0.0268
H2_O5_	0.26590	0.40394	0.37711	0.0268
H1_O9_	−0.16191	0.69906	0.16210	0.0216
H2_O9_	−0.13336	0.64516	0.37180	0.0216
H1_O6_	0.05410	0.45848	0.82379	0.0363

**Table 3:** Fractional coordinates at 100 K from laboratory X-rays. H atoms were constrained using a riding model with u_iso_ extended by a factor 1.2 of its neighboring non-H atom.

Atom	x	y	z	u_iso_ (Å^2^)
Fe^3+^(1)	0.44712(3)	0.700078(15)	0.32036(3)	0.00329(9)
Fe^2+^(2)	0.74909(3)	0.499263(15)	0.25324(3)	0.00432(9)
Fe^3+^(3)	0.94749(3)	0.702325(15)	0.81184(3)	0.00328(9)
P(1)	0.22520(6)	0.57863(3)	0.02106(5)	0.00384(15)
P(2)	0.72392(6)	0.57785(3)	0.52719(5)	0.00372(15)
P(3)	0.13792(6)	0.80377(3)	0.12925(5)	0.00321(15)
P(4)	0.63924(6)	0.69500(3)	0.13896(5)	0.00313(15)
O(1)	0.60509(15)	0.60030(8)	0.12305(15)	0.0055(5)
O(2)	0.04028(15)	0.76381(8)	0.18693(15)	0.0060(5)
O(3)	0.10500(15)	0.89858(8)	0.11500(15)	0.0053(5)
O(4)	0.57932(16)	0.77317(8)	0.48195(14)	0.0055(4)
O(5)	0.56225(15)	0.59929(8)	0.41183(15)	0.0055(4)
O(6)	0.06632(15)	0.60449(8)	−0.09539(15)	0.0061(5)
O(7)	0.30356(16)	0.63667(8)	0.14908(15)	0.0066(5)
O(8)	0.56779(16)	0.73350(8)	0.22446(15)	0.0070(5)
O(9)	0.81294(15)	0.70012(8)	0.22973(15)	0.0050(4)
O(10)	0.30685(15)	0.79620(8)	0.23661(14)	0.0048(4)
O(11)	0.09387(15)	0.77382(8)	−0.02750(14)	0.0050(4)
O(12)	0.93342(13)	0.55593(8)	0.23685(17)	0.0087(5)
O(13)	0.21307(17)	0.49793(8)	0.10164(16)	0.0087(5)
O(14)	0.79412(16)	0.63902(8)	0.65199(15)	0.0061(5)
O(15)	0.83661(11)	0.67851(7)	0.93809(15)	0.0068(5)
O(16)	0.56496(17)	0.44089(5)	0.26291(16)	0.0094(5)
O(17)	0.33467(15)	0.66954(7)	0.43550(15)	0.0071(5)
O(18)	0.32267(16)	0.55801(8)	−0.05033(15)	0.0062(5)
O(19)	0.72627(17)	0.49437(8)	0.60642(16)	0.0081(5)
O(20)	0.82174(16)	0.56083(8)	0.45385(15)	0.0061(5)
H1_O16_	0.47983	0.46119	0.22068	0.0113
H2_O16_	0.57951	0.39821	0.30884	0.0113
H1_O17_	0.32649	0.62190	0.45271	0.0085
H2_O17_	0.29809	0.70502	0.46412	0.0085
H1_O12_	0.94752	0.60480	0.25012	0.0105
H2_O12_	0.98925	0.52856	0.21735	0.0105
H1_O15_	0.86888	0.69868	1.02152	0.0082
H2_O15_	0.76146	0.64913	0.90348	0.0082
H1_O1_	0.52033	0.58753	0.05553	0.0066
H1_O3_	0.02027	0.91174	0.04792	0.0063
H1_O13_	0.20686	0.45668	0.14283	0.0104
H1_O19_	0.72748	0.45145	0.64716	0.0097

**Table 4:** Bond-lengths and bond-valence sums within FeO_6_ octahedra for the crystal structures obtained from the X-ray laboratory measurements with the lattice parameters given in the last two columns of Table 1 and atomic coordinates given in Table 2 and Table 3.

Temperature	300 K	100 K
Fe^2+^-O	Cyan	Cyan
	2.0654(16) Å x2	2.0620(15) Å 2.0751(15) Å
	2.1548(14) Å x2	2.1389(18) Å 2.1617(17) Å
	2.2127(15) Å x2	2.1847(13) Å 2.1919(13) Å
Average	2.1443 Å	2.1357 Å
Bond-valence sum	2.006	2.045
Fe^3+^-O	Beige 1.9240(16) Å 1.9605(15) Å 1.9738(15) Å 1.9937(15) Å 2.0110(14) Å 2.0842(14) Å	Orange/Beige 1.9422(13) Å/1.9174(13) Å 1.9505(13) Å/1.9586(13) Å 1.9810(20) Å/1.9732(12) Å 1.9832(13) Å/2.0000(19) Å 2.0176(14) Å/2.0073(14) Å 2.0602(19) Å/2.1205(17) Å
Average	1.9912 Å	1.9892 Å/1.9961 Å
Bond-valence sum	3.021	3.028/2.995

## Data Availability

The data used to support the findings of this study are available from the corresponding authors upon request.

## Supplementary Materials

The following supporting information can be downloaded at: https://www.mdpi.com/article/10.3390/ma15228059/s1, Video S1: Evolution of the crystal structure viewed along ***a****_LT_* axis, Video S2: Evolution of the crystal structure viewed along ***b****_LT_* axis and Video S3: Evolution of the crystal structure viewed along ***c****_LT_* axis.
